# Supplementary material for: Use of bio-information and communication technology shortens time to peak at a lower height of the epidemic curve: An alternative to flattening for countries with early COVID-19 outbreaks
Source: PLoS One. 2024 Apr 25;19(4):e0301669. doi: 10.1371/journal.pone.0301669 (PMC11045055; doi:10.1371/journal.pone.0301669)
Supplement: Attachment — Submitted filename: PO_Responsesf.docx [file pone.0301669.s001.docx]

**Authors’ Responses to the Reviewers’ Comments**

Reviewer #1: The manuscript aims to conceptualize the BICT-enabled epidemic control (BICTEC) and document its relationships with epidemic curve shaping and epidemic mitigation performance. The quality of the manuscript needs improvement by incorporating the following comments.

1. **Motivation, State of the art & Research questions - related to the objective of the work need to be presented in the Introduction Section**
   - *Response:* Thank you for pointing out these weaknesses. We've addressed this by adding a paragraph in the introduction section related to the study motivation and research questions.
2. **Related works need to be presented and compared.**
   - *Response:* Related works are added in the introduction and are compared with our study findings in the discussion section.
3. **Justify how the input data considered is sufficient for the proposed work.**
   - *Response:* Thank you for this comment. We recognize that the description of the input data is incomplete. We have, therefore, added several sentences in the Data Source subsection to sufficiently describe what the input data are. We have also added some citations to support the input data used.
4. **Justify how the input data are sufficient to address the proposed work objective.**
   - *Response:* We are not sure whether we have correctly understood this comment, which is given separately from the previous comment. The study objective was to determine whether the epidemic curve follows a flattened trajectory. For this objective, we have estimated key determinants of the epidemic curve and quantified their correlations. This justification is given under the conceptual framework subjection as well as under the analysis subsection which we have added per your comments. The objective related to curve shaping and epidemic mitigation performance is also described in the conceptual framework and analysis subsections.
5. **Justify how the considered input and analysis performed match the proposed work objective.**
   - *Response:* We have recognized the shortcomings in describing the relationship among the input, the analysis, and the study objective. Thus, we have added a subsection of analysis to link those elements.
6. **How the presented result analysis is considered correct/efficient compared to existing analysis in related works?**
   - *Response:* Again, we have recognized this weakness in comparing with existing works. Therefore, we have thoroughly revised the discussion section adding the previous work. We have also added a subsection as to whether the BICTEC performed better.

**Overall, the input considered, analysis performed & results presented are not sufficient to justify BICT-enabled epidemic control is efficient.**

- *Response:* Thank you for your valuable comments and reviews. Accordingly, we have revised the introduction, the methodology, and the discussion sections. Despite some limitations, we believe that our study findings on the BICTEC contribute to the literature.

Reviewer #2: Thank you for submitting this very interesting work. However, I would like to point out some improvements to be made.

1. **More quantitative information should be provided in the abstract.**
   - *Response:* Per your comments, we have added quantitative statistics on curve shaping and performance measures such as CFR and CI.
2. **Please provide the keywords after the abstract and write them alphabetically.**
   - *Response:* We've added five keywords after the abstract.

**In the introduction, please provide explanations about the COVID-19 epidemic and its control strategies worldwide. For this, you can use the following studies:**

*Environmental Health Engineering and Management Journal. 2022 Jan 10;9(1):41-53. <http://ehemj.com/article-1-901-en.html>

*Toxicology and Industrial Health. 2021 Jun;37(6):353-64. <https://doi.org/10.1177/07482337211013319>

- *Response:* Thank you for this comment. We've added a paragraph related to study motivation and existing work in the introduction section. Regarding the articles listed below that you have suggested we cite in our paper, they investigated strategies to identify factors affecting the management of the COVID pandemic in businesses such as hospitals and steel complexes. We apologize that we couldn’t cite your articles as a reference since these articles are not quite relevant to our topic. We hope this is acceptable.

3. **The discussion is brief. Please strengthen this section and compare the results of your study with studies conducted in other countries.**
   - *Response:* We appreciate your comments. We've added several paragraphs to compare our study results with previous work.
4. **The conclusion is very brief. Please indicate what implications the findings of this study hold for future research directions, policy-making, or public health strategies?**
   - *Response:* We appreciate your comments. We've added a paragraph to describe the implications of our study. Please also note that we have thoroughly revised the discussion section.
