# Supplementary material for: Use of bio-information and communication technology shortens time to peak at a lower height of the epidemic curve: An alternative to flattening for countries with early COVID-19 outbreaks
Source: PLoS One. 2024 Apr 25;19(4):e0301669. doi: 10.1371/journal.pone.0301669 (PMC11045055; doi:10.1371/journal.pone.0301669)
Supplement: Attachment — Submitted filename: BITEX_PO_Response2f.docx [file pone.0301669.s002.docx]

Responses to the reviewer’s comments.

Thank you for your feedback. Our responses have been included below each comment for your convenience. If you have any further concerns or questions, please do not hesitate to let us know.

Again, thank you for your valuable comments.

Reviewer #1: The quality of the manuscript needs to be improved by addressing the following comments,
- Research Problem, State of the art works, & Research Questions need to be provided in Introduction section

**Authors Responses**: Thank you for the comments.

To elucidate these important concepts, we have revised the fourth paragraph of the introduction section as below. Additionally, we have included references related to the Susceptible-Infectious-Recovered (SIR) model to provide a contrast with our study. Please also note that our research hypotheses are already presented in the methodology under the subheading of analysis.

“Of particular interest is to investigate whether the BICT contact tracing shapes the epidemic curve differently that the traditional flattened one. While numerous studies have explored various shapes of epidemic curves, most of them are variants of the flattened curve that can be explained based on the Susceptible-Infectious-Recovered (SIR) model or its analogs [12-14]. Few studies have proposed an alternative shape that is distinct from the flattened curve. A recent study published in The Lancet described two different epidemic trajectories: one where peak infection occurs sooner at a shorter height, and another with a flattened normal trajectory [2]. However, the study did not establish a connection between these trajectories and different strategies for epidemic control. The former was simply linked to effective social distancing, while the latter was linked to typical social distancing. Furthermore, the study did not elaborate on what constitutes effective social distancing. Therefore, the objective of this study is to investigate the relationship between epidemic trajectories and epidemic control strategies. The results of this study would inform the design of effective strategies to contain initial surges of cases in epidemic outbreaks in a highly-connected modern society.”

- Related works need to be discussed. At least 12-15 works need to be discussed in terms of Problem, solution recommended, results obtained and research gap.

**Authors Responses:** In our discussion, we made sure to cite related works at least 15 times. Our primary focus was to highlight our findings and provide support for them. Thus, we structured the discussion in a way that our results would support our hypotheses: 1) country-specific epidemic curves follow an alternative shape compared to the flattened one; 2) epidemic control performance (CI and CFR) is associated with this shape. Throughout this process, we incorporated literature evidence to support our results.

To be frank, we found it challenging to fully understand the reviewer's comment regarding the recommended discussion of 12-15 works in terms of problem, solution recommended, results obtained, and research gap. We are not aware of any specific journal guidelines on this recommendation. However, we believe that our discussion adequately addresses the problem (the inadequacy of the flattened curve in reflecting the outcomes of modern epidemic control strategies), the solution recommended (the BICTEC shapes the epidemic curve to peak sooner at a shorter height), the results obtained (our study findings), and the research gap (how our study fills a gap in the literature as well as our study limitations).

- Summary of the related works and scope for improvements need to be provided.

**Authors Responses:** We found it challenging to incorporate your recommendation regarding "the provision of a summary of the related works and scope for improvements." We are not aware of any journal guidelines that require the provision of a summary of related works. Our paper did not aim to provide such a summary. Instead, our focus was on presenting empirical evidence for an alternative epidemic trajectory. We therefore highlighted existing research on epidemic control strategies and their limitations in the introduction. Additionally, in the discussion section, we compared our study findings with previous research to contextualize our results. We used statements to maintain a logical flow throughout the paper. We are concerned that providing a summary of the related works may lengthen our research paper unnecessarily and disrupt its logical flow.

As for the scope for improvements, we acknowledge that there is always room for further research and advancements in this field. We have therefore already mentioned the limitations along with potential directions for future research. We hope this clarifies our approach to introducing related works and addressing areas for improvement.

- Novelty of the proposed methodology need to be justified.

**Authors Responses:** While novelty exists concerning the concept of BICT-based contact tracing, there is no novelty associated with the methodology. In fact, the methodology used to estimate the epidemic curve characteristics is well-documented

- Comparison with existing work/methodology need to be provided for justifying the benefits of proposed work/methodology.

**Authors Responses:** As for the comparison with existing work, it has been addressed in the discussion section. Regarding the comparison with existing methodologies, it falls outside the scope of our study as outlined in our specific aims. However, the methodology employed to estimate the epidemic curve is thoroughly described in the methodology section, along with relevant citations.
